# An Improved Initial Alignment Method Based on SE_2_(3)/EKF for SINS/GNSS Integrated Navigation System with Large Misalignment Angles

**DOI:** 10.3390/s24092945

**Published:** 2024-05-06

**Authors:** Jin Sun, Yuxin Chen, Bingbo Cui

**Affiliations:** 1 College of Internet of Things, Nanjing University of Posts and Telecommunications, Nanjing 210003, China; sunjin@njupt.edu.cn; 2 State Key Laboratory of Ocean Engineering, Shanghai Jiao Tong University, Shanghai 200240, China; 3 Portland Institute, Nanjing University of Posts and Telecommunications, Nanjing 210003, China; p21000213@njupt.edu.cn; 4 Key Laboratory of Modern Agricultural Equipment and Technology, Ministry of Education and Jiangsu Province, Jiangsu University, Zhenjiang 212013, China; 5 School of Agricultural Engineering, Jiangsu University, Zhenjiang 212013, China

**Keywords:** large misalignment angles, initial alignment, strap-down inertial navigation system (SINS), global navigation satellite system (GNSS), three-dimension special Euclidean group and extended Kalman filter (SE_2_(3)/EKF), Lie group, SINS/GNSS integrated navigation system

## Abstract

This paper proposes an improved initial alignment method for a strap-down inertial navigation system/global navigation satellite system (SINS/GNSS) integrated navigation system with large misalignment angles. Its methodology is based on the three-dimensional special Euclidean group and extended Kalman filter (SE_2_(3)/EKF) and aims to overcome the challenges of achieving fast alignment under large misalignment angles using traditional methods. To accurately characterize the state errors of attitude, velocity, and position, these elements are constructed as elements of a Lie group. The nonlinear error on the Lie group can then be well quantified. Additionally, a group vector mixed error model is developed, taking into account the zero bias errors of gyroscopes and accelerometers. Using this new error definition, a GNSS-assisted SINS dynamic initial alignment algorithm is derived, which is based on the invariance of velocity and position measurements. Simulation experiments demonstrate that the alignment method based on SE_2_(3)/EKF can achieve a higher accuracy in various scenarios with large misalignment angles, while the attitude error can be rapidly reduced to a lower level.

## 1. Introduction

The initial alignment is a crucial component of the strap-down inertial navigation system (SINS) and is assessed based on its speed and accuracy [1]. Currently, there are two main categories of traditional alignment methods based on misalignment angles—small misalignment angles linear alignment and large misalignment angles nonlinear alignment [2]. Linear alignment models and linear filtering algorithms for small misalignment angles are well established, while research on the alignment problems for large misalignment angles has mainly focused on nonlinear models and nonlinear filtering algorithms [3]. However, these approaches can lead to errors in model linearization, increased computational complexity, and reduced filtering accuracy for large misalignment angles. In the 21st century, modern warfare has demonstrated the significance of high-tech conditions, where precision is a key consideration, alongside speed. Consequently, it is essential to find ways to achieve fast and highly accurate initial alignment under large misalignment angles, for applications like weapon launchers and guided weapons that require emergency mobile transfer. Addressing this urgent problem is crucial from both a system performance perspective in modern warfare and from practical alignment environments, data utilization, and engineering software development [4,5,6]. Hence, studying the linearized uniform initial alignment algorithm without coarse alignment at any misalignment angles is of paramount importance and offers significant theoretical and engineering benefits.

The current most popular mode of navigation is the SINS/Global navigation satellite system (GNSS) integrated navigation model [7,8]. Satellite navigation offers both high accuracy and low cost. In the SINS/GNSS integrated navigation system, GNSS not only improves navigation accuracy by correcting inertial navigation information, but also provides initial position and velocity information for SINS [9]. However, the GNSS cannot directly provide attitude information. In recent years, nonlinear system state estimation has gained attention in the field of inertial navigation for combined navigation and initial alignment. This includes methods such as the extended Kalman filter (EKF) [10], unscented Kalman filter (UKF) [11,12], particle filter (PF) [13], cubature Kalman filter (CKF) [14], unscented particle filter (UPF) [15], distributed Kalman filter (DKF) [16], or a combination of multiple nonlinear filters [17,18]. Among these, the traditional EKF has two drawbacks. Firstly, it requires high accuracy in the initial value of the system state. If the initial value is based on the actual situation, the filter may struggle to converge. Secondly, the EKF can lead to inconsistency. When a new observation is received, the EKF calculates the covariance matrix of the current state based on the linearization of the previous state. However, the actual value of the covariance matrix may not align with this calculated value. Compared to the EKF, the UKF is more practical. However, both the traditional UKF and EKF require prior statistics on known system noise and measurement noise. In practical applications, the accuracy of the filter is inevitably affected by environmental limitations and algorithm problems, leading to a decrease in accuracy or even divergence.

In the traditional EKF framework used for the GNSS/SINS integrated navigation system, state errors such as position and velocity are defined only by considering differences in size, completely ignoring differences in direction. This oversight can lead to inconsistent definitions of state error coordinate systems. In recent years, a new filter has been developed based on Lie groups and manifold theory. Its core idea is to define the states and errors such as attitude, velocity, and position in the group space, rather than in the traditional Euclidean space. According to the multiplicative closure property of Lie groups and the affine property between Lie algebras, a more compact dynamic equation of position and coordinate state errors, considering attitude errors, has been redesigned to meet the requirement that the error transfer matrix or measurement matrix remain unchanged or slowly change and to achieve the independence of F or H matrix and state estimation. Therefore, it can be unchanged, also known as invariant EKF. The error definition corresponding to the invariant EKF has good self-consistency, which effectively overcomes the defect that the traditional EKF is too dependent on the initial value, and has better convergence and consistency of filtering [19]. It is more rigorous, in theory, and has been well applied in inertial navigation fields such as robot attitude estimation, simultaneous localization and mapping (SLAM), and visual odometers (VOs) [20].To address this issue, this paper adopts the invariant EKF concept. It defines states and errors, such as attitude, velocity, and position, in group space. Based on this approach, new inertial navigation error equations and measurement update methods are deduced. The main contributions of this paper can be summarized as follows: (1) Constructing the attitude, velocity, and position states as three-dimensional special Euclidean group (SE_2_(3))/EKF elements, which takes into account the zero bias errors of gyroscopes and accelerometers. This facilitates the formation of a mixed group vector error. The alignment method based on SE_2_(3)/EKF filtering has demonstrated faster convergence speed, higher accuracy, and greater computational efficiency than the traditional EKF. (2) Attaining the accurate position of the vehicle during the alignment process is crucial for improving the alignment accuracy of the system. Moreover, the position alignment aids in directly transitioning the vehicle to the autonomous navigation stage after the GNSS-aided alignment. Consequently, it enhances the vehicle’s applicability, by eliminating the need to obtain the vehicle’s position again at the end of the alignment. Accurate and real-time position alignment can improve the attitude alignment accuracy of the system.

The paper follows this outline—Section 2 establishes the inertial navigation error equation under the Lie group framework. Section 3 presents the filtering model for the SINS/GNSS integrated navigation system based on SE_2_(3)/EKF. The simulation experiment was conducted in Section 4. Finally, Section 5 concludes.

## 2. Inertial Navigation Error Equation under the Lie Group Framework

SE_2_(3)/EKF does not simply use the difference between the estimated state and the real state as the error, but more carefully considers the consistency of the coordinate frame of the state definition and provides a more rigorous and compact mathematical form by redefining the error in Lie group space. In order to describe the state error, a lie group state error model including attitude, velocity, and position is designed and the gyro bias error and accelerometer bias error are still defined in the traditional vector space.

The related Cartesian reference coordinate frames used in this study are defined as follows [21]:

(1) *b*-frame: Body coordinate system, with its three axes pointing to the right–front–up (R-F-U) of the carrier, respectively, denoted as $x_{b}y_{b}z_{b}$;
(2) *n*-frame: It indicates the navigation frame and it is the frame used by the SINS to calculate the navigation parameters, denoted as Eastward–Northward–Upward (E-N-U);
(3) *e*-frame: Earth coordinate system, with its origin at the geocenter. The *x*-axis is the intersection of the geocenter pointing to the prime meridian and the equator, the *z*-axis is the geocenter pointing to the north pole, and the *y*-axis forms a right-handed coordinate system with the *x*-axis and *z*-axis, denoted as $x_{e}y_{e}z_{e}$;
(4) *i*-frame: Inertial coordinate system. It is a non-rotating coordinate system in inertial space, denoted as $x_{i}y_{i}z_{i}$.

**Figure 1:**
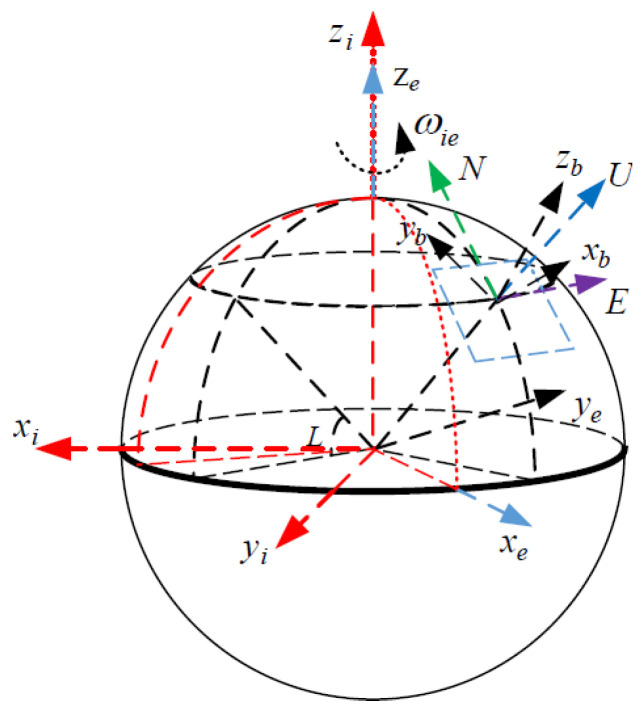
Schematic diagram of the coordinate system.

The schematic diagram of the coordinate system mentioned above is shown in Figure 1. $\omega_{ie}$ is the angular velocity of the Earth’s rotation and L is the latitude of the SINS.

### 2.1. SINS Navigation Differential and Error Equations

Selecting the E-N-U geographic coordinate system as the navigation reference coordinate system for the SINS, the attitude differential equation using the n-frame as the reference frame is as follows [22]:

(1) $${\dot{C}}_{b}^{n}=C_{b}^{n}(\omega_{ib}^{b}\times )-[(\omega_{ie}^{n}+\omega_{en}^{n})\times ]C_{b}^{n}$$

where $C_{b}^{n}$ denotes the attitude matrix of the *b*-frame relative to the *n*-frame, $\omega_{ib}^{b}$ is the angular velocity of the b-frame relative to the *i*-frame, $\omega_{ie}^{n}$ is the rotational angular velocity of the Earth, $\omega_{ie}^{n}={\left[\begin{array}{ccc} 0 & \omega_{ie}\cos L & \omega_{ie}sinL \end{array}\right]}^{T}$, $\omega_{en}^{n}$ is the angular velocity generated by the relative motion of the n-frame to the *e*-frame, $\omega_{en}^{n}={\left[\begin{array}{ccc} -\frac{v_{N}}{R_{M}+h} & \frac{v_{E}}{R_{N}+h} & \frac{v_{E}}{R_{N}+h}\tan L \end{array}\right]}^{T}$, and h is the geographical altitude. $R_{M}$ is the principal radius of curvature along the meridional section, $R_{N}$ is the principal radius of curvature along the prime-vertical normal section, $v^{n}$ is the velocity in the n-frame, and $v^{n}={\left[\begin{array}{ccc} v_{E} & v_{N} & v_{U} \end{array}\right]}^{T}$. $v_{E}$, $v_{N}$, and $v_{U}$ are the eastern, northern, and upward velocity, respectively. × denotes the conversion of vectors into oblique symmetric matrices.

The differential equation for attitude error can be derived as follows [22]:

(2) $${\dot{\phi }}^{n}=\phi^{n}\times \omega_{in}^{n}+\delta \omega_{in}^{n}-C_{b}^{n}\delta \omega_{ib}^{b}$$

where ϕ is attitude error, $\omega_{in}^{n}=\omega_{ie}^{n}+\omega_{en}^{n}$, $\delta \omega_{in}^{n}$ is the calculation error, $\delta \omega_{in}^{n}=\delta \omega_{ie}^{n}+\delta \omega_{en}^{n}$, $\delta \omega_{ie}^{n}={\left[\begin{array}{ccc} 0 & -\omega_{ie}\sin L\cdot \delta L & \omega_{ie}\cos L\cdot \delta L \end{array}\right]}^{T}=M_{1}\delta p^{n}$, $M_{1}=\left[\begin{array}{ccc} 0 & 0 & 0\\ -\omega_{ie}\sin L & 0 & 0\\ \omega_{ie}\cos L & 0 & 0 \end{array}\right]$, and $\delta \omega_{ib}^{b}$ is the measurement error of the gyroscope.

(3) $$\delta \omega_{en}^{n}=\left[\begin{array}{c} \frac{-\delta v_{N}}{R_{M}+h}+\frac{v_{N}\delta h}{{\left(R_{M}+h\right)}^{2}}\\ \frac{\delta v_{E}}{R_{N}+h}-\frac{v_{E}\delta h}{{\left(R_{N}+h\right)}^{2}}\\ \frac{\tan L\cdot \delta v_{E}}{R_{N}+h}+\frac{v_{E}\sec^{2}L\cdot \delta L}{R_{N}+h}-\frac{v_{E}\tan L\cdot \delta h}{{\left(R_{N}+h\right)}^{2}} \end{array}\right]=M_{av}\delta v^{n}+M_{2}\delta p^{n}$$

where $M_{av}=\left[\begin{array}{ccc} 0 & -\frac{1}{R_{M}+h} & 0\\ \frac{1}{R_{N}+h} & 0 & 0\\ \frac{\tan L}{R_{N}+h} & 0 & 0 \end{array}\right]$, $M_{2}=\left[\begin{array}{ccc} 0 & 0 & \frac{v_{N}}{{\left(R_{M}+h\right)}^{2}}\\ 0 & 0 & -\frac{v_{E}}{{\left(R_{N}+h\right)}^{2}}\\ \frac{v_{E}\sec^{2}L}{R_{N}+h} & 0 & -\frac{v_{E}\tan L}{{\left(R_{N}+h\right)}^{2}} \end{array}\right]$, $p^{n}={\left[\begin{array}{ccc} L & \lambda & h \end{array}\right]}^{T}$, λ is the longitude, $\delta p^{n}={\left[\begin{array}{ccc} \delta L & \delta \lambda & \delta h \end{array}\right]}^{T}$ is the position error, and δL, δλ, and δh are the latitude error, longitude error, and height errors, respectively. $\delta v^{n}$ is the velocity error in the n-frame, $\delta v^{n}={\left[\begin{array}{ccc} \delta v_{E} & \delta v_{N} & \delta v_{U} \end{array}\right]}^{T}$, and $\delta v_{E}$, $\delta v_{N}$, and $\delta v_{U}$ are the eastern, northern, and upward velocity errors, respectively.

The velocity differential equation defined under the local navigation system is as follows [18]:

(4) $${\dot{v}}^{n}=C_{b}^{n}f^{b}-(2\omega_{ie}^{n}+\omega_{en}^{n})\times v^{n}+g^{n}$$

where $f^{b}$ is the specific force measured using the accelerometer, $2\omega_{ie}^{n}\times v^{n}$ is the Coriolis acceleration caused by the motion of the carrier and the rotation of the Earth, $\omega_{en}^{n}\times v^{n}$ is the centripetal acceleration caused by the movement of the carrier towards the ground, $g^{n}$ is gravitational acceleration, and $-(2\omega_{ie}^{n}+\omega_{en}^{n})\times v^{n}+g^{n}$ is collectively referred to as harmful acceleration.

The corresponding differential equation for velocity error is as follows [23]:

(5) $$\delta {\dot{v}}^{n}=f^{n}\times \phi^{n}+v^{n}\times (2\delta \omega_{ie}^{n}+\delta \omega_{en}^{n})-(2\omega_{ie}^{n}+\omega_{en}^{n})\times \delta v^{n}+C_{b}^{n}\delta f^{b}+\delta g^{n}$$

where $f^{n}$ is the projection of the specific force vector in the n-frame, $\delta f^{b}$ is the measurement error of the accelerometer, and $\delta g^{n}$ is the gravitational acceleration error.

The position differential equation defined under the local navigation system is as follows [23]:

(6) $${\dot{p}}^{n}={\left[\begin{array}{ccc} \dot{L} & \dot{\lambda } & \dot{h} \end{array}\right]}^{T}=\left[\begin{array}{ccc} 0 & \frac{1}{R_{M}+h} & 0\\ \frac{\sec L}{R_{N}+h} & 0 & 0\\ 0 & 0 & 1 \end{array}\right]v^{n}=M_{pv}v^{n}$$

where $M_{pv}=\left[\begin{array}{ccc} 0 & \frac{1}{R_{M}+h} & 0\\ \frac{\sec L}{R_{N}+h} & 0 & 0\\ 0 & 0 & 1 \end{array}\right]$.

The corresponding differential equation for positional error is as follows [23]:

(7) $$\begin{array}{ll} \delta {\dot{p}}^{n} & ={\left[\begin{array}{ccc} \delta \dot{L} & \delta \dot{\lambda } & \delta \dot{h} \end{array}\right]}^{T}\\ & =\left[\begin{array}{ccc} 0 & \frac{1}{R_{M}+h} & 0\\ \frac{\sec L}{R_{N}+h} & 0 & 0\\ 0 & 0 & 1 \end{array}\right]\delta v^{n}+\left[\begin{array}{ccc} 0 & 0 & \frac{-v_{N}}{{\left(R_{M}+h\right)}^{2}}\\ \frac{v_{E}\sec L\tan L}{R_{N}+h} & 0 & \frac{-v_{E}\sec L}{{\left(R_{N}+h\right)}^{2}}\\ 0 & 0 & 0 \end{array}\right]\delta p^{n}\\ & =M_{pv}\delta v^{n}+M_{pp}\delta p^{n} \end{array}$$

The error model for gyroscopes and accelerometers is as follows:

(8) $$\{\begin{array}{c} {\hat{\omega }}_{ib}^{b}=\omega_{ib}^{b}+\varepsilon^{b}+w_{g}\\ {\hat{f}}^{b}=f^{b}+\nabla^{b}+w_{a} \end{array}$$

where ${\hat{\omega }}_{ib}^{b}$ and ${\hat{f}}^{b}$ are actual measured values of gyroscopes and accelerometers, respectively. $\varepsilon^{b}$ is the random constant drift of the gyroscopes, $\nabla^{b}$ is the random constant bias of the accelerometers, and ${\dot{\varepsilon }}^{b}=0$, ${\dot{\nabla }}^{b}=0$. $w_{g}$, and $w_{a}$ are the Gaussian white noises of the accelerometers and gyroscopes, respectively.

### 2.2. Left Invariant Error Equation of Inertial Navigation in the Lie Group Framework

In the Lie group framework, the n-frame is chosen as the projection coordinate system for the SINS/GNSS integrated system. The attitude, velocity, and position of the n-frame are defined as a group, χ, [24] as follows:

(9) $$\chi =\left[\begin{array}{ccc} C_{b}^{n} & v^{n} & p^{n}\\ 0_{1\times 3} & 1 & 0\\ 0_{1\times 3} & 0 & 1 \end{array}\right]$$

where $C_{b}^{n}\in SO(3)$, SO(3) is a three-dimensional special orthogonal group, $0_{1\times 3}$ is a zero matrix with one row and three columns, and χ∈SE(3) is a more concise group that includes elements of attitude, velocity, and position.

By using the properties of Lie groups [25], we can obtain the following:

(10) $$\chi^{-1}=\left[\begin{array}{ccc} C_{n}^{b} & -v^{b} & -p^{b}\\ 0_{1\times 3} & 1 & 0\\ 0_{1\times 3} & 0 & 1 \end{array}\right]$$

where $\chi^{-1}$ denotes the inverse matrix of χ and, similarly, $\chi^{-1}\in SE(3)$.

If χ represents the true state and $\hat{\chi }$ represents the nominal state, the error of the attitude, velocity, and position states is defined as η∈SE(3). The error in Lie group space can be classified into two types—left invariance, $\eta ={\hat{\chi }}^{-1}\chi$, and right invariance, $\eta =\chi {\hat{\chi }}^{-1}$. Research has shown that left invariance can achieve the invariance or gradual change of the measurement matrix, while the observations of GNSS theoretically belong to the category of left invariant observations [25]; therefore, this paper focuses on the left invariant error for processing.

Based on Equations (1) and (2), the specific expression for the left invariant error is given by:

(11) $$\eta ={\hat{\chi }}^{-1}\chi =\left[\begin{array}{ccc} {\hat{C}}_{n}^{b} & -{\hat{v}}^{b} & -{\hat{p}}^{b}\\ 0_{1\times 3} & 1 & 0\\ 0_{1\times 3} & 0 & 1 \end{array}\right]\left[\begin{array}{ccc} C_{b}^{n} & v^{n} & p^{n}\\ 0_{1\times 3} & 1 & 0\\ 0_{1\times 3} & 0 & 1 \end{array}\right]=\left[\begin{array}{ccc} {\hat{C}}_{n}^{b}C_{b}^{n} & {\hat{C}}_{n}^{b}v^{n}-{\hat{v}}^{b} & {\hat{C}}_{n}^{b}p^{n}-{\hat{p}}^{b}\\ 0_{1\times 3} & 1 & 0\\ 0_{1\times 3} & 0 & 1 \end{array}\right]$$

The errors corresponding to the attitude, velocity, and position under the defined Lie group are as follows:

(12) $${\hat{C}}_{n}^{b}C_{b}^{n}=Exp(\phi^{b})=\exp(\phi^{b}\times )$$

(13) $$J\rho_{v}^{b}={\hat{C}}_{n}^{b}v^{n}-{\hat{v}}^{b}={\hat{C}}_{n}^{b}v^{n}-{\hat{C}}_{n}^{b}{\hat{v}}^{n}=-{\hat{C}}_{n}^{b}\delta v^{n}$$

(14) $$J\rho_{p}^{b}={\hat{C}}_{n}^{b}p^{n}-{\hat{p}}^{b}={\hat{C}}_{n}^{b}p^{n}-{\hat{C}}_{n}^{b}{\hat{p}}^{n}=-{\hat{C}}_{n}^{b}\delta p^{n}$$

where $\phi^{b}$ is the attitude error angles, Exp(⋅) represents the mapping between Lie algebras and Lie groups, and exp(⋅) represents exponentiation. $\exp(\phi^{b}\times )=I_{3}+\frac{\sin\theta }{\theta }(\phi^{b}\times )+\frac{1-\cos\theta }{\theta^{2}}{\left(\phi^{b}\times \right)}^{2}$ and θ=|ϕ|. J is the Jacobian matrix of the Rodriguez formula and $J=\sum_{n=0}^{\infty }\frac{1}{(n+1)!}(\phi^{b}\times )=I_{3}+\frac{1-\cos\theta }{\theta^{2}}(\phi^{b}\times )+\frac{\theta -\sin\theta }{\theta^{3}}{\left(\phi^{b}\times \right)}^{2}$. $J\rho_{v}^{b}$ is the new definition of velocity error and $J\rho_{p}^{b}$ is the new definition of positional error.

From the error form defined in Equation (11), we can observe that the velocity and position errors defined in the Lie group framework include attitude terms. This takes into account the differences in numerical magnitude and direction between true values and estimates. Therefore, the error definition is more reasonable and concise compared to the traditional vector space method of differencing, which solves the problem of inconsistent benchmarks for defining velocity error states.

According to the characteristics of Lie groups and Lie algebras, the left invariant error satisfies the following [26,27]:

(15) $$\eta =\left[\begin{array}{ccc} \exp(\phi^{n}\times ) & J\rho_{v}^{b} & J\rho_{p}^{b}\\ 0_{1\times 3} & 1 & 0\\ 0_{1\times 3} & 0 & 1 \end{array}\right]$$

The new attitude error equation is derived as follows:

(16) $$\begin{array}{ll} \frac{d}{dt}\left({\hat{C}}_{n}^{b}C_{b}^{n}\right) & ={\dot{\hat{C}}}_{n}^{b}C_{b}^{n}+{\hat{C}}_{n}^{b}{\dot{C}}_{b}^{n}\\ & =\left[{\hat{C}}_{n}^{b}({\hat{\omega }}_{in}^{n}\times )-({\hat{\omega }}_{ib}^{b}\times ){\hat{C}}_{n}^{b}\right]C_{b}^{n}+{\hat{C}}_{n}^{b}\left[C_{b}^{n}(\omega_{ib}^{b}\times )-(\omega_{in}^{n}\times )C_{b}^{n}\right]\\ & ={\hat{C}}_{n}^{b}({\hat{\omega }}_{in}^{n}\times )C_{b}^{n}-({\hat{\omega }}_{ib}^{b}\times ){\hat{C}}_{n}^{b}C_{b}^{n}+{\hat{C}}_{n}^{b}C_{b}^{n}(\omega_{ib}^{b}\times )-{\hat{C}}_{n}^{b}(\omega_{in}^{n}\times )C_{b}^{n}\\ & \approx {\hat{C}}_{n}^{b}\left[({\hat{\omega }}_{in}^{n}-\omega_{in}^{n})\times \right]C_{b}^{n}-\left[(\omega_{ib}^{b}+\delta \omega_{ib}^{b})\times \right]\left[I_{3}+(\phi^{b}\times )\right]+\left[I_{3}+(\phi^{b}\times )\right](\omega_{ib}^{b}\times )\\ & \approx \delta \omega_{in}^{n}\times \left[I_{3}+(\phi^{b}\times )\right]+(\phi^{b}\times \omega_{ib}^{b})\times -\delta \omega_{ib}^{b}\times \\ & \approx \delta \omega_{in}^{n}\times +(\phi^{b}\times \omega_{ib}^{b})\times -\delta \omega_{ib}^{b}\times \end{array}$$

where ${\hat{C}}_{n}^{b}C_{b}^{n}\approx I_{3}+(\phi^{b}\times )$, $\delta \omega_{in}^{n}={\hat{\omega }}_{in}^{n}-\omega_{in}^{n}$, $\delta \omega_{ib}^{b}={\hat{\omega }}_{ib}^{b}-\omega_{ib}^{b}$, and the second-order small quantities $\left(\delta \omega_{in}^{n}\times )(\phi^{b}\times \right)$ and $\left(\delta \omega_{ib}^{b}\times )(\phi^{b}\times \right)$ are neglected in the process of formula derivation.

The new velocity error equation is derived as follows:

(17) $$\begin{array}{ll} \frac{d}{dt}\left(J\rho_{v}^{b}\right) & =-{\dot{\hat{C}}}_{n}^{b}\delta v^{n}-{\hat{C}}_{n}^{b}\delta {\dot{v}}^{n}\\ & =\left[{\hat{C}}_{n}^{b}({\hat{\omega }}_{in}^{n}\times )-({\hat{\omega }}_{ib}^{b}\times ){\hat{C}}_{n}^{b}\right]\delta v^{n}\\ & -{\hat{C}}_{n}^{b}\left[f^{n}\times \phi^{n}+v^{n}\times (2\delta \omega_{ie}^{n}+\delta \omega_{en}^{n})-(2\omega_{ie}^{n}+\omega_{en}^{n})\times \delta v^{n}+C_{b}^{n}\delta f^{b}+\delta g^{n}\right]\\ & \approx -({\hat{\omega }}_{ib}^{b}\times )J\rho_{v}^{b}-\delta f^{b}+\phi^{b}\times f^{b}-C_{n}^{b}\delta g^{n}+(C_{n}^{b}\omega_{ie}^{n})\times J\rho_{v}^{b}-C_{n}^{b}(v^{n}\times )(\delta \omega_{ie}^{n}+\delta \omega_{in}^{n}) \end{array}$$

where $\delta f^{b}={\hat{f}}^{b}-f^{b}$, $\delta \omega_{ie}^{n}={\hat{\omega }}_{ie}^{n}-\omega_{ie}^{n}$, and $\delta g^{n}={\hat{g}}^{n}-g^{n}$.

The new position error equation is derived as follows:

(18) $$\begin{array}{ll} \frac{d}{dt}\left(J\rho_{p}^{b}\right) & =-{\dot{\hat{C}}}_{n}^{b}\delta p^{n}-{\hat{C}}_{n}^{b}\delta {\dot{p}}^{n}\\ & =-\left[{\hat{C}}_{n}^{b}({\hat{\omega }}_{in}^{n}\times )-({\hat{\omega }}_{ib}^{b}\times ){\hat{C}}_{n}^{b}\right]\delta p^{n}-{\hat{C}}_{n}^{b}(M_{pv}\delta v^{n}+M_{pp}\delta p^{n})\\ & =({\hat{\omega }}_{ib}^{b}\times ){\hat{C}}_{n}^{b}\delta p^{n}-{\hat{C}}_{n}^{b}({\hat{\omega }}_{in}^{n}\times )\delta p^{n}-{\hat{C}}_{n}^{b}M_{pv}\delta v^{n}-{\hat{C}}_{n}^{b}M_{pp}\delta p^{n}\\ & \approx -({\hat{\omega }}_{ib}^{b}\times )J\rho_{p}^{b}+J\rho_{v}^{b}-C_{n}^{b}(p^{n}\times )\delta \omega_{en}^{n}+(C_{n}^{b}\omega_{ie}^{n})\times J\rho_{p}^{b} \end{array}$$

By substituting Equations (13) and (14) into $\delta \omega_{ie}^{n}$ and $\delta \omega_{in}^{n}$, we can obtain the following equations:

(19) $$\delta \omega_{ie}^{n}=M_{1}\delta p^{n}=-M_{1}{\hat{C}}_{b}^{n}J\rho_{p}^{b}$$

(20) $$\delta \omega_{in}^{n}=(M_{1}+M_{2})\delta p^{n}+M_{av}\delta v^{n}=-(M_{1}+M_{2}){\hat{C}}_{b}^{n}J\rho_{p}^{b}-M_{av}{\hat{C}}_{b}^{n}J\rho_{v}^{b}$$

Finally, the error equations of SINS can be written as follows:

(21) $$\frac{d}{dt}\left({\hat{C}}_{n}^{b}C_{b}^{n}\right)=-C_{n}^{b}(M_{1}+M_{2}){\hat{C}}_{b}^{n}J\rho_{p}^{b}-C_{n}^{b}M_{av}{\hat{C}}_{b}^{n}J\rho_{v}^{b}-\omega_{ib}^{b}\times \phi^{b}-(\varepsilon^{b}+w_{g})$$

(22) $$\begin{array}{ll} \frac{d}{dt}\left(J\rho_{v}^{b}\right) & =C_{n}^{b}(v^{n}\times )(2M_{1}+M_{2})C_{b}^{n}J\rho_{p}^{b}+\left[C_{n}^{b}(v^{n}\times )M_{av}C_{b}^{n}-(\omega_{ib}^{b}\times )-(C_{n}^{b}\omega_{ie}^{n})\times \right]J\rho_{v}^{b}\\ & -f^{b}\times \phi^{b}-C_{n}^{b}\delta g^{n}-(\nabla^{b}+w_{a}) \end{array}$$

(23) $$\frac{d}{dt}\left(J\rho_{p}^{b}\right)=\left[C_{n}^{b}(p^{n}\times )M_{2}C_{b}^{n}-(\omega_{ib}^{b}\times )+(C_{n}^{b}\omega_{ie}^{n})\times \right]J\rho_{p}^{b}+\left[I_{3}+C_{n}^{b}(p^{n}\times )M_{av}C_{b}^{n}\right]J\rho_{v}^{b}$$

## 3. Filtering Model Based on SE_2_(3)/EKF

### 3.1. State Equation Based on SE_2_(3)

If the state vector is X and the system noise vector is W, then the state equation based on SE_2_(3) is as follows [28]:

(24) $$\dot{X}=FX+GW$$

where F is the state transition matrix, G is the system noise allocation matrix, and $E[WW^{T}]=Q$.

(25) $$X={\left[\begin{array}{lllll} {\left(\phi^{b}\right)}^{T} & {\left(J\rho_{v}^{b}\right)}^{T} & {\left(J\rho_{p}^{b}\right)}^{T} & {\left(\varepsilon^{b}\right)}^{T} & {\left(\nabla^{b}\right)}^{T} \end{array}\right]}^{T}$$

(26) $$F=\left[\begin{array}{lllll} -{\hat{\omega }}_{ib}^{b}\times & -{\hat{C}}_{n}^{b}M_{av}{\hat{C}}_{b}^{n} & {\hat{C}}_{n}^{b}(M_{1}+M_{2}) & -I_{3} & 0_{3}\\ -{\hat{f}}^{b}\times & {\hat{C}}_{n}^{b}({\hat{v}}^{n}\times )M_{av}{\hat{C}}_{b}^{n}-{\hat{\omega }}_{ib}^{b}\times -{\hat{C}}_{n}^{b}({\hat{\omega }}_{ie}^{n}\times ){\hat{C}}_{b}^{n} & -{\hat{C}}_{n}^{b}({\hat{v}}^{n}\times )(2M_{1}+M_{2}) & 0_{3} & -I_{3}\\ 0_{3} & -M_{pv}{\hat{C}}_{b}^{n} & M_{pv} & 0_{3} & 0_{3}\\ 0_{3} & 0_{3} & 0_{3} & 0_{3} & 0_{3}\\ 0_{3} & 0_{3} & 0_{3} & 0_{3} & 0_{3} \end{array}\right]$$

(27) $$G=\left[\begin{array}{ll} -I_{3} & 0_{3}\\ 0_{3} & -I_{3}\\ 0_{3} & 0_{3}\\ 0_{3} & 0_{3}\\ 0_{3} & 0_{3} \end{array}\right]$$

(28) $$W=\left[\begin{array}{c} w_{g}\\ w_{a} \end{array}\right]$$

where $I_{3}$ is a 3×3 unit vector and $0_{3}$ is a 3×3 zero matrix.

### 3.2. Measurement Equation Based on SE_2_(3)

The GNSS can provide position and velocity information in integrated navigation as measurements for Kalman filtering, to suppress the divergence of inertial navigation calculation results. Therefore, the measurement equation based on the SE_2_(3) can be written as follows [29]:

(29) $$Z=\left[\begin{array}{c} v_{SINS}^{n}-v_{GNSS}^{n}\\ p_{SINS}^{n}-p_{GNSS}^{n} \end{array}\right]=HX+V$$

where $v_{SINS}^{n}$ and $v_{GNSS}^{n}$ are the velocity of the SINS and the GNSS in the n-frame, respectively. $p_{SINS}^{n}$ and $p_{GNSS}^{n}$ are the position of the SINS and the GNSS in the n-frame, respectively. H is the measurement matrix and V is the white noise for velocity measurement and position measurement of satellite receivers.

(30) $$H=\left[\begin{array}{llllll} {\hat{v}}^{n} & -I_{3} & 0_{3} & 0_{3} & 0_{3} & 0_{3}\\ 0_{3} & 0_{3} & 0_{3} & 0_{3} & 0_{3} & 0_{3} \end{array}\right]$$

(31) $$V=\left[\begin{array}{c} V_{v}\\ V_{p} \end{array}\right]$$

where $V_{v}$ and $V_{p}$ are the white noise for satellite receiver velocity measurement and position measurement, respectively.

### 3.3. SE_2_(3)/EKF Algorithm

Assuming the sampling interval of IMU is Δt and the discretized state transition matrix is Φ, then the SE_2_(3)/EKF algorithm is as follows [30]:

(1) One-step state prediction
  (32) $${\hat{X}}_{k,k-1}=\Phi_{k,k-1}{\hat{X}}_{k-1}$$
(2) State estimation
  (33) $${\hat{X}}_{k}={\hat{X}}_{k,k-1}+\delta {\hat{X}}_{k}$$
(3) State estimation error
  (34) $$\delta {\hat{X}}_{k}=K_{k}(Z_{k}-H_{k}{\hat{X}}_{k,k-1})$$
(4) Filter gain
  (35) $$K_{k}=P_{k,k-1}H_{k}^{T}{\left[H_{k}P_{k,k-1}H_{k}^{T}+R_{k}\right]}^{-1}$$
(5) One-step prediction mean square error
  (36) $$P_{k,k-1}=\Phi_{k,k-1}P_{k-1}\Phi_{k,k-1}^{T}+Q_{k-1}$$
(6) Estimating mean square error
  (37) $$P_{k}=(I-K_{k}H_{k})P_{k,k-1}{\left(I-K_{k}H_{k}\right)}^{T}+K_{k}R_{k}K_{k}^{T}$$
  where ${\hat{X}}_{k,k-1}$ is the state one-step prediction matrix, $\Phi_{k,k-1}$ is the one-step transition matrix from time $t_{k-1}$ to time $t_{k}$, and $\Phi_{k,k-1}=I+F\Delta t$. ${\hat{X}}_{k-1}$ is the state estimation matrix at time $t_{k-1}$, $\delta {\hat{X}}_{k}$ is the state estimation error matrix at time $t_{k}$, $K_{k}$ is the filtering gain matrix at time $t_{k}$, $Z_{k}$ is the measurement matrix at time $t_{k}$, $P_{k,k-1}$ is the one step prediction mean square error matrix, $R_{k}$ is the measurement noise variance matrix, $Q_{k-1}$ is the system noise variance matrix at time $t_{k-1}$, $Q_{k-1}=G_{k-1}(q_{k-1}\Delta t)G_{k-1}^{T}$, $G_{k-1}$ is the discretized system noise allocation matrix at time $t_{k-1}$, and q is the variance intensity matrix corresponding to the system noise matrix W. $P_{k}$ is the estimated mean square error matrix.

## 4. Simulation Results

**Figure 2:**
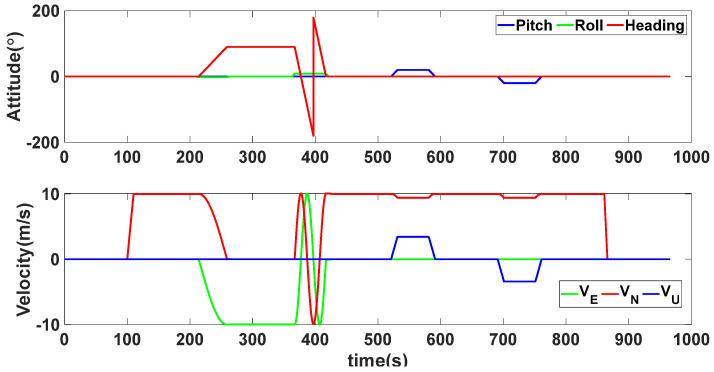
The dynamic characteristics of the vehicle during the experiment.

**Figure 3:**
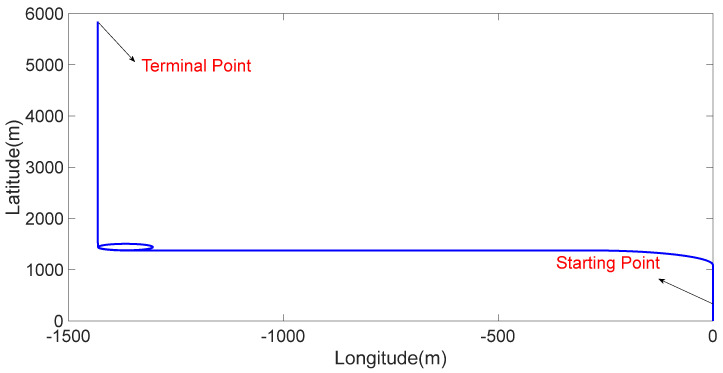
The trajectory of the vehicle during the experiment.

This section presents the simulation experiments conducted to verify the validity and feasibility of the proposed method. The specific parameters of the inertial sensor used in the simulation experiments are as follows: the constant drift and random walk of the gyroscopes are represented by values $0.03^{\circ }/h$ and $0.001^{\circ }/\sqrt{h}$, respectively. The constant bias and random bias of the accelerometers are represented by values 100 μg and 10 μg, respectively. The data output frequency is 200 Hz and the calculation period of SINS is 5 ms. The performance parameters of the GNSS are as follows: the velocity measurement accuracy is represented by a value of 0.1 m/s, the position measurement accuracy is represented by a value of 1 m, and the data output frequency is represented by a value of 1 Hz. The initial position is set as follows: $118.786365^{\circ }\;E$, $32.057313^{\circ }\;N$, and the height is 0 m. The initial velocity is represented by a value of ${\left[\begin{array}{ccc} 0 & 0 & 0 \end{array}\right]}^{T}\;m/s$. The simulation time is 900 s. The state and trajectory of the vehicle are illustrated in Figure 2 and Figure 3, respectively.

To fully demonstrate the performance of the proposed algorithm, contrast experiments of SE_2_(3)/EKF and the EKF algorithm, as well as position alignment experiments, will be designed. These experiments will compare the traditional EKF with the SE_2_(3)/EKF algorithm proposed in this paper, using three different initial misalignment angle scenarios. The performance of various algorithms will be evaluated to verify the advantages of the SE_2_(3)/EKF algorithm.

### 4.1. Experiment 1

**Figure 4:**
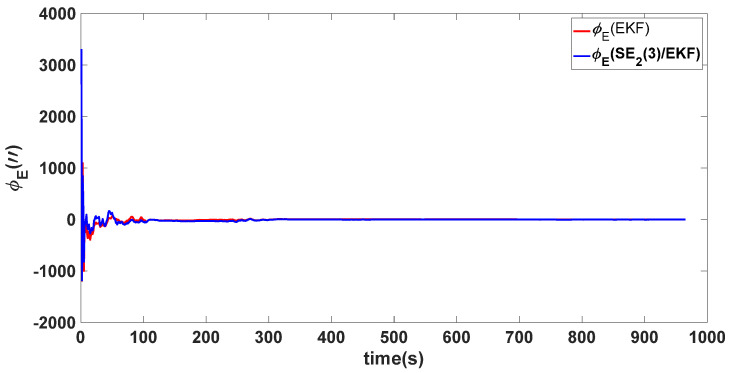
Eastward attitude angle error of misalignment angles (${\left[\begin{array}{ccc} 1^{\circ } & -1^{\circ } & 40^{\circ } \end{array}\right]}^{T}$).

**Figure 5:**
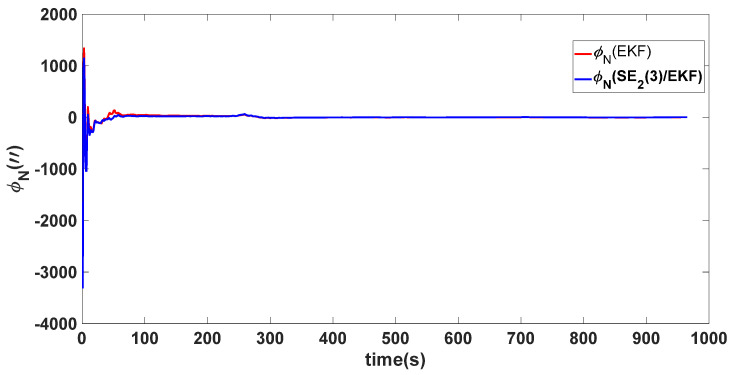
Northward attitude angle error of misalignment angles (${\left[\begin{array}{ccc} 1^{\circ } & -1^{\circ } & 40^{\circ } \end{array}\right]}^{T}$).

**Figure 6:**
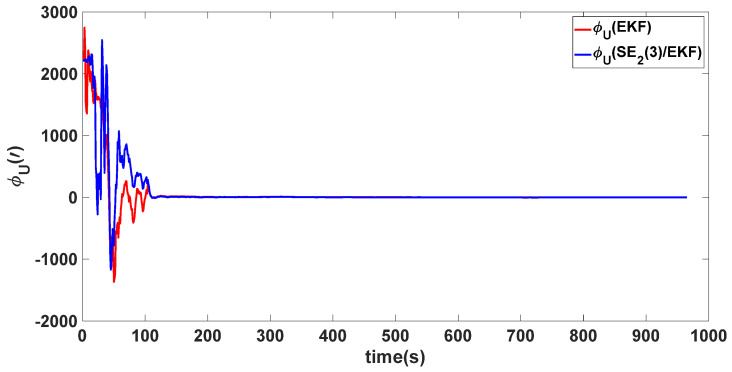
Upward attitude angle error of misalignment angles (${\left[\begin{array}{ccc} 1^{\circ } & -1^{\circ } & 40^{\circ } \end{array}\right]}^{T}$).

**Figure 7:**
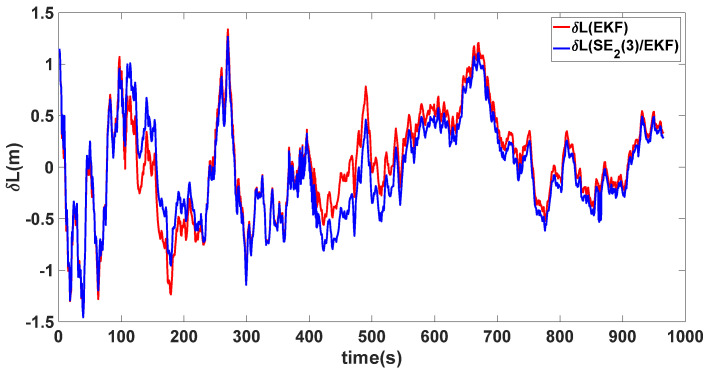
Latitude alignment error of misalignment angles (${\left[\begin{array}{ccc} 1^{\circ } & -1^{\circ } & 40^{\circ } \end{array}\right]}^{T}$).

**Figure 8:**
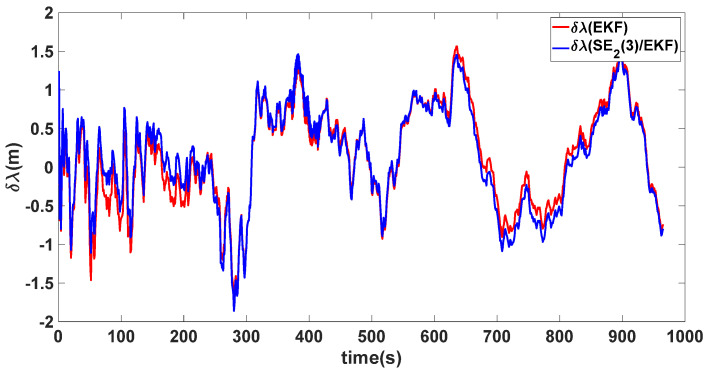
Longitude alignment error of misalignment angles (${\left[\begin{array}{ccc} 1^{\circ } & -1^{\circ } & 40^{\circ } \end{array}\right]}^{T}$).

**Figure 9:**
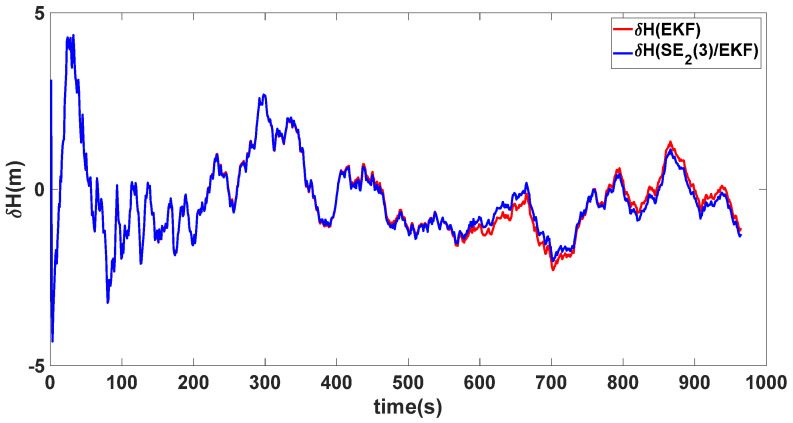
Height alignment error of misalignment angles (${\left[\begin{array}{ccc} 1^{\circ } & -1^{\circ } & 40^{\circ } \end{array}\right]}^{T}$).

In this experiment, the misalignment angles in the three directions are sequentially set ${\left[\begin{array}{ccc} 1^{\circ } & -1^{\circ } & 40^{\circ } \end{array}\right]}^{T}$. The mean and variance of the attitude error and position error are recorded in Table 1 for the 50 s leading up to the end of alignment. The attitude angle error are as shown in Figure 4, Figure 5 and Figure 6, and the position alignment error are as shown in Figure 7, Figure 8 and Figure 9.

Based on the comprehensive information from the chart, both methods can converge the attitude angles to a relatively stable range in this scenario. The figure shows that the time taken to converge the horizontal attitude angles estimated by the two methods is not significantly different; both methods converge quickly. However, the convergence time for the heading angles is longer. The data provided in Table 1 indicate that the attitude angle accuracy of the two methods after alignment is relatively close. Overall, under the misalignment angles set in Experiment 1, both methods perform equally. This is because the initial misalignment angles are small at this time and the nonlinearity of the traditional EKF model is not severe. Additionally, defining the velocity error as the direct difference between the true value and the estimated value is considered reasonable, allowing both alignment methods to converge quickly.

### 4.2. Experiment 2

**Figure 10:**
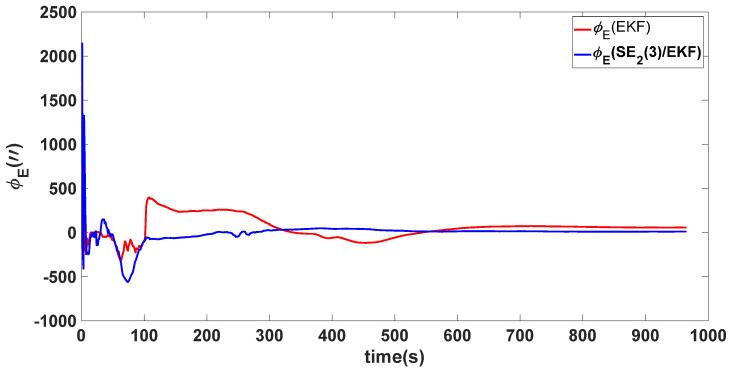
Eastward attitude angle error of misalignment angles (${\left[\begin{array}{ccc} 1^{\circ } & -1^{\circ } & 95^{\circ } \end{array}\right]}^{T}$).

**Figure 11:**
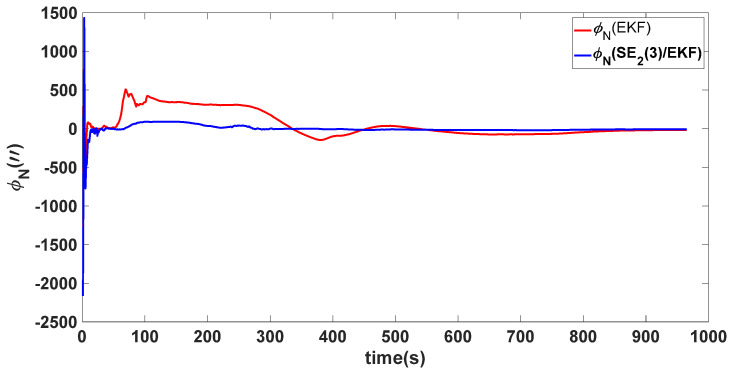
Northward attitude angle error of misalignment angles (${\left[\begin{array}{ccc} 1^{\circ } & -1^{\circ } & 95^{\circ } \end{array}\right]}^{T}$).

**Figure 12:**
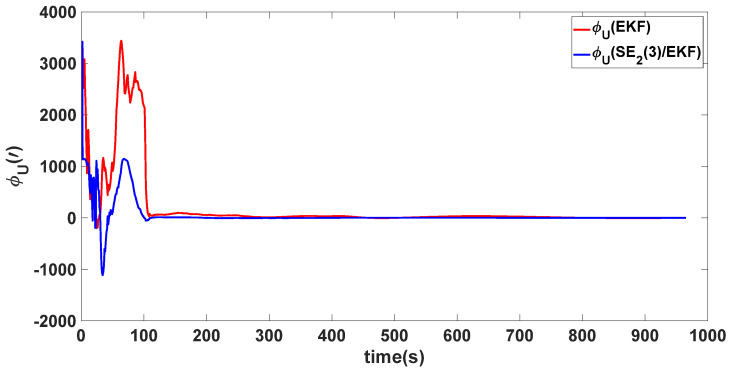
Upward attitude angle error of misalignment angles (${\left[\begin{array}{ccc} 1^{\circ } & -1^{\circ } & 95^{\circ } \end{array}\right]}^{T}$).

**Figure 13:**
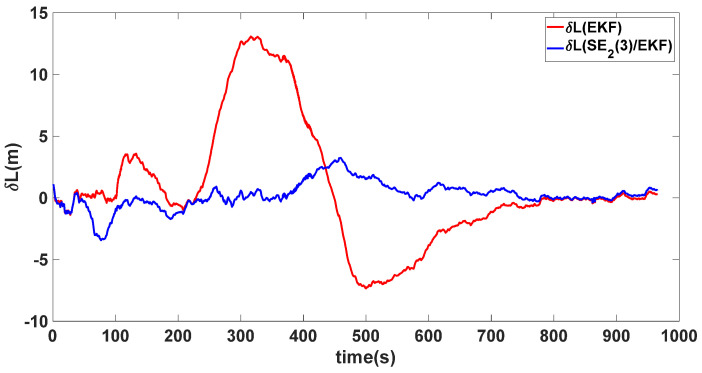
Latitude alignment error of misalignment angles (${\left[\begin{array}{ccc} 1^{\circ } & -1^{\circ } & 95^{\circ } \end{array}\right]}^{T}$).

**Figure 14:**
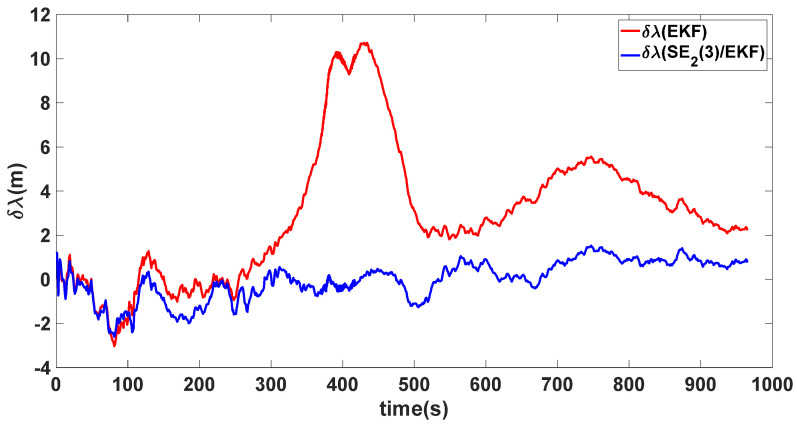
Longitude alignment error of misalignment angles (${\left[\begin{array}{ccc} 1^{\circ } & -1^{\circ } & 95^{\circ } \end{array}\right]}^{T}$).

**Figure 15:**
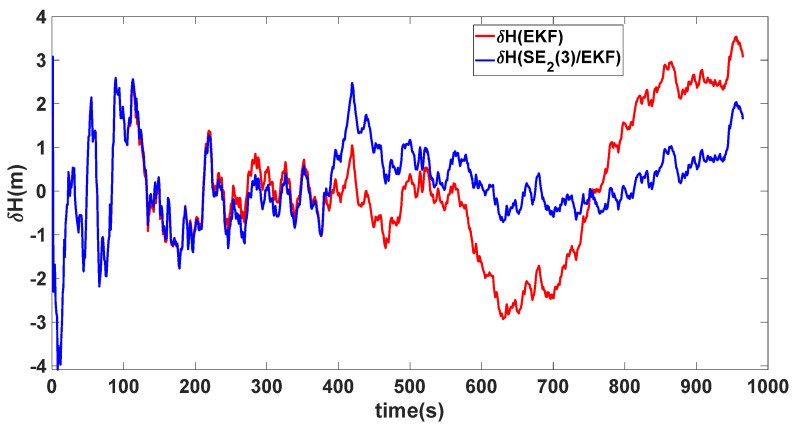
Height alignment error of misalignment angles (${\left[\begin{array}{ccc} 1^{\circ } & -1^{\circ } & 95^{\circ } \end{array}\right]}^{T}$).

In this experiment, the misalignment angles in the three directions are sequentially set ${\left[\begin{array}{ccc} 1^{\circ } & -1^{\circ } & 95^{\circ } \end{array}\right]}^{T}$. The mean and variance of the attitude error and position error are recorded in Table 2 for the 50 s leading up to the end of alignment. The attitude angle error are as shown in Figure 10, Figure 11 and Figure 12, and the position alignment error are as shown in Figure 13, Figure 14 and Figure 15.

From the figures, it can be observed that, due to further increasing the initial heading angle error, the nonlinearity of the traditional model is enhanced. At this point, the EKF method still uses a linear error differential equation error model, disregarding the influence of high-order system terms. As a result, significant model errors occur, leading to a slower convergence speed in attitude compared to the SE_2_(3)/EKF method. According to Table 2, the convergence accuracy of the EKF method is lower than that of SE_2_(3)/EKF. The SE_2_(3)/EKF method redefines errors more rigorously within the Lie group framework, resulting in a better performance for larger misalignment angles.

### 4.3. Experiment 3

**Figure 16:**
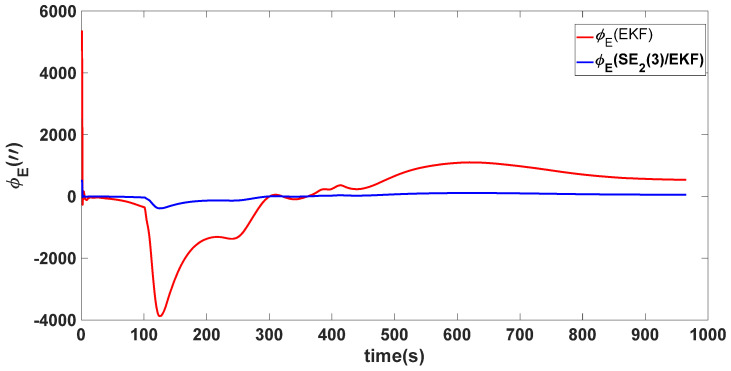
Eastward attitude angle error of misalignment angles (${\left[\begin{array}{ccc} 20^{\circ } & 20^{\circ } & 165^{\circ } \end{array}\right]}^{T}$).

**Figure 17:**
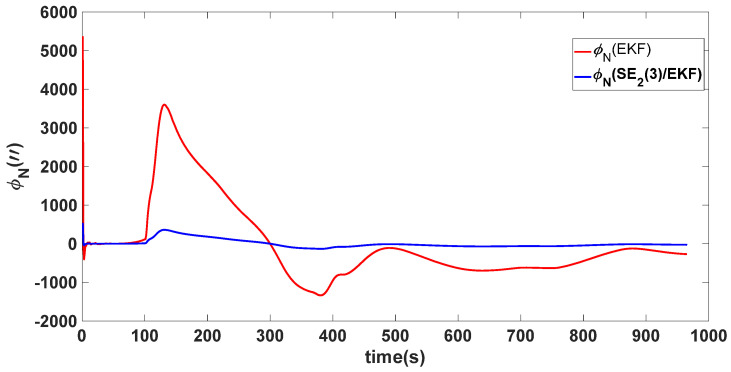
Northward attitude angle error of misalignment angles (${\left[\begin{array}{ccc} 20^{\circ } & 20^{\circ } & 165^{\circ } \end{array}\right]}^{T}$).

**Figure 18:**
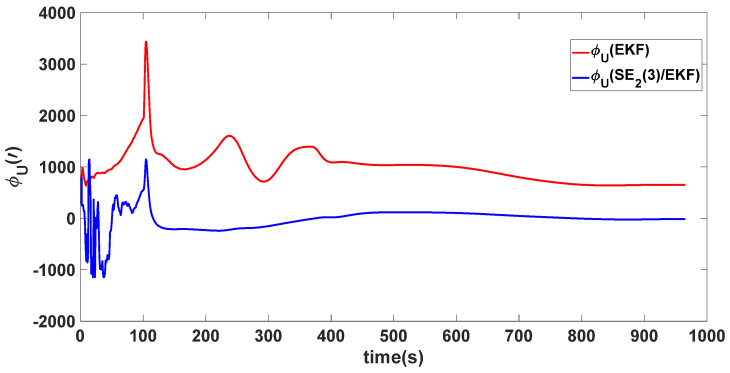
Upward attitude angle error of misalignment angles (${\left[\begin{array}{ccc} 20^{\circ } & 20^{\circ } & 165^{\circ } \end{array}\right]}^{T}$).

**Figure 19:**
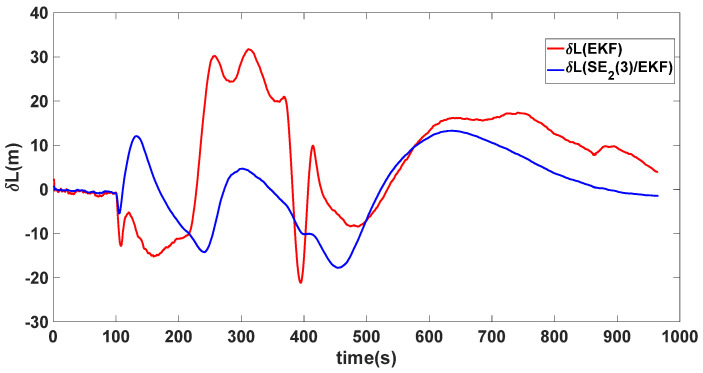
Latitude alignment error of misalignment angles (${\left[\begin{array}{ccc} 20^{\circ } & 20^{\circ } & 165^{\circ } \end{array}\right]}^{T}$).

**Figure 20:**
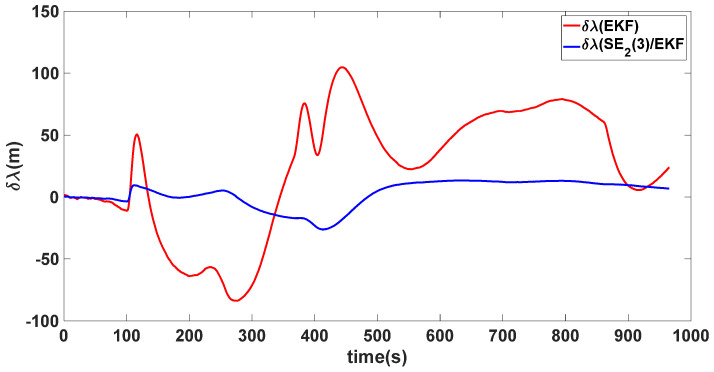
Longitude alignment error of misalignment angles (${\left[\begin{array}{ccc} 20^{\circ } & 20^{\circ } & 165^{\circ } \end{array}\right]}^{T}$).

**Figure 21:**
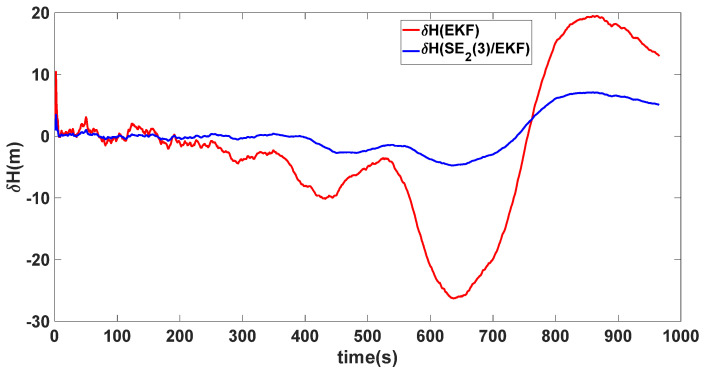
Height alignment error of misalignment angles (${\left[\begin{array}{ccc} 20^{\circ } & 20^{\circ } & 165^{\circ } \end{array}\right]}^{T}$).

In this experiment, the misalignment angles in the three directions are sequentially set ${\left[\begin{array}{ccc} 20^{\circ } & 20^{\circ } & 165^{\circ } \end{array}\right]}^{T}$. The mean and variance of the attitude error and position error are recorded in Table 3 for the 50 s leading up to the end of alignment. The attitude angle error are as shown in Figure 16, Figure 17 and Figure 18, and the position alignment error are as shown in Figure 19, Figure 20 and Figure 21.

The figure shows that, when the misalignment angles in three directions reach a severe level, the nonlinearity of the model becomes extremely severe. Additionally, defining the velocity error as a direct subtraction becomes even more unreasonable. Consequently, the traditional EKF algorithm is no longer able to converge, while the SE_2_(3)/EKF method still converges relatively quickly. According to Table 3, it is evident that, in this scenario, the attitude angle error calculated by SE_2_(3)/EKF can still maintain a level close to Experiment 1 and Experiment 2, further demonstrating the stable characteristics of the SE_2_(3)/EKF alignment method.

## 5. Conclusions

This paper proposed a novel initial alignment method for the SINS/GNSS integrated navigation system with large misalignment angles based on SE_2_(3)/EKF. The left invariant SE_2_(3)/EKF adopts a group-vector mixed error model, which allows the linear state error based on Lie algebra to effectively capture the nonlinear error on the Lie group. This compatibility with the linear assumption of EKF filtering results in a higher precision dynamic model, improved measurement update accuracy, and better performance in dynamic initial alignment, especially when considering attitude error. Simulation experiments demonstrated that the EKF method can achieve attitude convergence in scenarios with small initial misalignment angles. On the other hand, the alignment method based on SE_2_(3)/EKF can converge to higher accuracy in the different large misalignment angle scenarios simulated in this paper, quickly reducing attitude errors to a lower level.

In future research, we focus on studying a more reasonable difference resistance adaptive filtering method within the framework of SE_2_(3). This will enable us to adapt to complex GNSS observation environments or high dynamic scenes of carriers, expanding the application scenario of this algorithm. Additionally, we aim to conduct real-time vehicle experiments to validate the proposed methodology for practical use.

## Figures and Tables

**Table 1:** Mean and variance of attitude error and position error (${\left[\begin{array}{ccc} 1^{\circ } & -1^{\circ } & 40^{\circ } \end{array}\right]}^{T}$).

Algorithm	Attitude and Position Error	Mean	Variance
SE_2_(3)/EKF	Pitch Error	−0.7871″	0.1214
	Roll Error	1.2537″	0.0926
	Heading Error	0.0595′	0.0087
	Latitude Error	0.4381 m	0.0412
	Longitude Error	−0.2522 m	0.0201
	Height Error	−0.3418 m	0.0603
EKF	Pitch Error	0.9947″	0.1216
	Roll Error	1.9856″	0.0945
	Heading Error	−0.3037′	0.0107
	Latitude Error	0.4882 m	0.0415
	Longitude Error	−0.3065 m	0.0203
	Height Error	−0.5243 m	0.0616

**Table 2:** Mean and variance of attitude error and position error (${\left[\begin{array}{ccc} 1^{\circ } & -1^{\circ } & 95^{\circ } \end{array}\right]}^{T}$).

Algorithm	Attitude and Position Error	Mean	Variance
SE_2_(3)/EKF	Pitch Error	11.0881″	0.2950
	Roll Error	−7.7115″	0.1303
	Heading Error	−1.0848′	0.0259
	Latitude Error	0.2507 m	0.1201
	Longitude Error	0.8113 m	0.0552
	Height Error	1.6058 m	0.1191
EKF	Pitch Error	57.2824″	0.3147
	Roll Error	−17.7575″	0.1366
	Heading Error	−2.5052′	0.0445
	Latitude Error	0.5569 m	0.1238
	Longitude Error	2.3491 m	0.0612
	Height Error	3.1618 m	0.1307

**Table 3:** Mean and variance of attitude error and position error (${\left[\begin{array}{ccc} 20^{\circ } & 20^{\circ } & 165^{\circ } \end{array}\right]}^{T}$).

Algorithm	Attitude and Position Error	Mean	Variance
SE_2_(3)/EKF	Pitch Error	13.641″	0.4387
	Roll Error	17.0″	1.2976
	Heading Error	4.5116′	0.2122
	Latitude Error	−1.8307 m	0.0204
	Longitude Error	2.2376 m	0.1855
	Height Error	4.1076 m	0.1372
EKF	Pitch Error	$5.4181\times 10^{2}$″	0.4905
	Roll Error	$-2.5211\times 10^{2}$″	2.2026
	Heading Error	$-38.9656\times 10^{2}$′	0.1851
	Latitude Error	5.3826 m	0.1123
	Longitude Error	22.3550 m	0.6989
	Height Error	16.1874 m	0.1525

## Data Availability

The data presented in this study are available on request from the corresponding author.

## Abbreviations

The abbreviations used in this manuscript are as follows:

SINS	Strap-down Inertial Navigation System
GNSS	Global Navigation Satellite System
SE	Set Euclidean
EKF	Extended Kalman Filter
UKF	Unscented Kalman Filter
PF	Particle Filter
CKF	Cubature Kalman Filter
UPF	Unscented Particle Filter
